# 3D Reconstruction Model of an Extra-Abdominal Desmoid Tumor: A Case Study

**DOI:** 10.3389/fbioe.2020.00518

**Published:** 2020-06-12

**Authors:** Franco Marinozzi, Francesco Carleo, Simone Novelli, Marco Di Martino, Giuseppe Cardillo, Lea Petrella, Fabiano Bini

**Affiliations:** ^1^Department of Mechanical and Aerospace Engineering, “Sapienza” University of Rome, Rome, Italy; ^2^Thoracic Surgery Unit, San Camillo-Forlanini Hospital, Rome, Italy; ^3^Department of Methods and Models for Economics, Territory and Finance (MEMOTEF), “Sapienza” University of Rome, Rome, Italy

**Keywords:** desmoid tumor, thoracic surgery, diagnostic imaging, 3D reconstruction, 3D printing

## Abstract

In recent years, three-dimensional reconstruction (3DR) models have become a standard tool in several medical fields such as education, surgical training simulation, patient–doctor communication, and surgical planning. Postoncologic reconstructive surgery in thoracic diseases might benefit from 3DR models; however, limited data on this application have been published worldwide. In this paper, the aim was to report our experience with 3DR modeling to determine resection and plan the surgical reconstruction in a patient with a desmoid tumor of the chest wall. For a better understanding of the case study, we describe all the steps from acquiring computed tomography (CT) scans to the final 3D rendering. A 68-year-old, non-smoking man presented at our outpatient department with painless swelling of the right anterobasal chest wall. A thorax–abdomen–brain CT scan revealed homogenous solid tissue with a dense mass measuring 80 mm × 62 mm. The final 3D model was evaluated by the surgical team (three medical doctors), who found the model to be powerful. Based on the results and the accuracy of the model, the multidisciplinary team decided that the tumor was resectable. Consequently, a surgical plan based on the 3D model was developed to perform chest wall reconstruction after radical resection. The patient underwent right anterolateral thoracotomy at the seventh intercostal space, which confirmed the CT scan findings and revealed infiltration of the serratus muscle and medial portion of the diaphragm. A radical tumor en bloc resection with chest wall and diaphragm resection was performed. The full-thickness chest wall and diaphragm defects were reconstructed using two separate biological patches of a porcine dermal collagen implant (Permacol™ Surgical Implant). Postoperative X-ray revealed unremarkable findings; the patient had an uneventful recovery and was discharged 6 days after surgery. This case study illustrates that 3DR models enable a personalized approach to the treatment of desmoid tumors. Therefore, this approach should be developed further and studied systematically.

## Introduction

Desmoid tumors (also called aggressive fibromatosis) are benign, slow-growing fibroblastic neoplasms with no metastatic potential but a propensity for local recurrence, even after complete surgical resection. Unlike the intra-abdominal desmoids that are frequently associated with familial adenomatous polyposis (FAP) and are characterized by a more locally aggressive behavior with a tendency to severe relapse requiring multimodal treatment, extra-abdominal desmoid tumors can benefit from surgical resection (Molloy et al., 2012; Howard and Pollock, 2016). In this instance, where surgery is required, a high recurrence rate has been described (Ozger et al., 2007). Given the high rate of infiltration of desmoid lesions into muscle fibers, “clear margin” resections are not always achievable, potentially explaining the recurrence. In contrast, wide resections with negative margins have been shown to result in lower recurrence rates (Gronchi et al., 2003). Chest wall tumor resections are some of the most challenging surgeries for thoracic surgeons and can lead to significant postoperative pulmonary dysfunction. Such tumors are best approached in a multidisciplinary fashion to adequately address defects and optimize postoperative outcomes. The use of three-dimensional reconstruction (3DR) technology with automated and manual segmentation is revolutionizing many sectors and is becoming a standard tool in several areas of medicine, including education, surgical training, and patient–doctor communication (Bici et al., 2018; Bergquist et al., 2019; Guachi et al., 2019). Apart from obvious “visual” superiority, a 3D model also provides a valuable visual experience thanks to its pre- and intraoperative manipulation. Due to the difficult management of desmoid tumors involving the chest wall, the application of 3DR modeling for decision-making regarding the potential of curative resection may be of interest. Here, we describe the use of 3DR modeling to determine resectability and plan surgical reconstruction in a patient presenting with a desmoid tumor of the chest wall.

## Case Study Presentation

**Figure 1:**
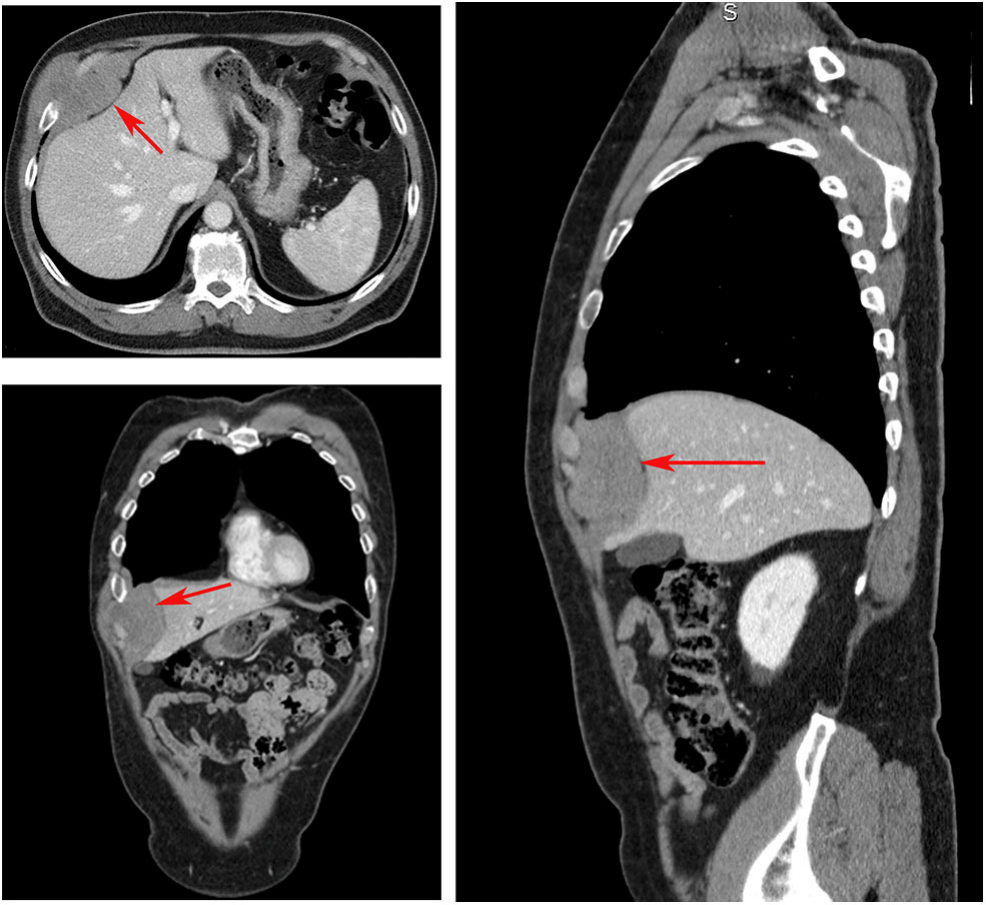
Computed tomography scan showing a homogenous, solid tissue, dense mass (red arrows) of 80 mm × 62 mm, in the anterobasal region of the right hemothorax involving the anterior arch of the seventh, eighth, and ninth ribs and their cartilages.

A 68-year-old non-smoking man presented at our outpatient department with painless swelling of the right anterobasal chest wall noticed for 2 months while taking a shower. There was no history of weight loss or trauma. His hemogram and liver function tests were within normal limits. A thorax–abdomen–brain computed tomography (CT) scan revealed homogenous solid tissue (dense mass of 80 mm × 62 mm), in the anterobasal region of the right hemothorax involving the anterior arch of the seventh, eighth, and ninth ribs and their cartilages (Figure 1). The mass had polycyclic contours, projecting into the chest wall touching the liver with no infiltration. Fine-needle aspiration biopsy led to the diagnosis of a spindle cell tumor consistent with an extra-abdominal desmoid tumor. The patient underwent right anterolateral thoracotomy at the seventh intercostal space which, confirmed the CT scan findings and revealed infiltration of the serratus muscle and the medial portion of the diaphragm. The tumor was resected with a wide full-thickness chest wall resection of the affected ribs (7th, 8th, 9th, and 10th), diaphragm, and serratus muscle. The resected diaphragm and chest wall were reconstructed using two separate biological patches of a porcine dermal collagen implant (Permacol™ Surgical Implant) (Limura and Giordano, 2017). The resected specimen was 130 mm × 120 mm × 60 mm (with a mass of 90 mm). Histopathology of the radically resected specimen showed a circumscribed, fibrotic tumor consistent with an extra-abdominal desmoid tumor, involving soft tissues of the chest wall, ribs, and diaphragm. Postoperative X-ray was unremarkable, and the patient had an uneventful recovery and was discharged 6 days after surgery. Written informed consent was obtained from the patient for the publication of this case report.

## Materials and Methods

**Figure 2:**
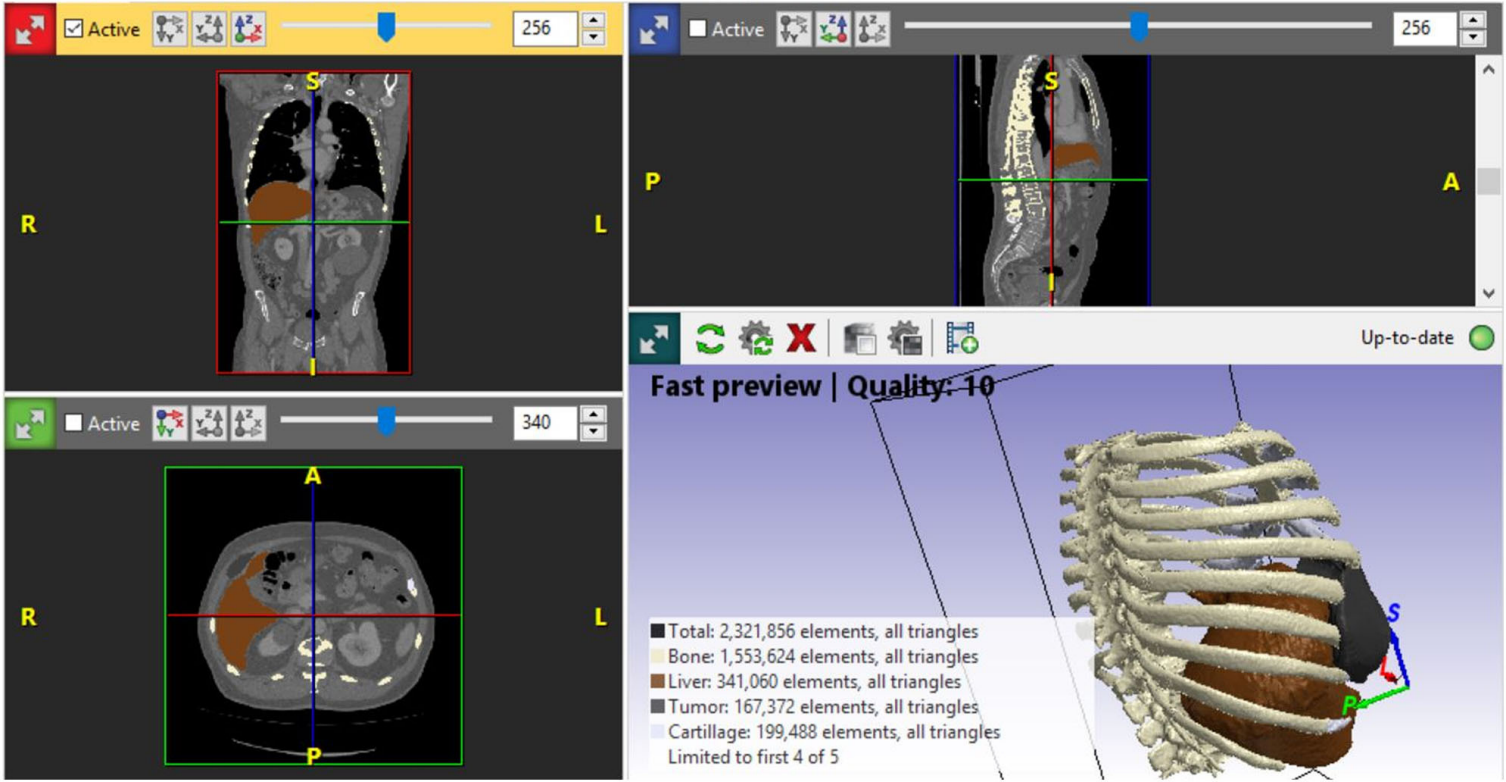
Simpleware ScanIP software snapshot applied for segmentation of the organ, ribs, cartilages, and tumor. ScanIP is the software used for comprehensively processing three-dimensional image data from a computed tomography scan. The software provides powerful image visualization, analysis, segmentation, and quantifications tools.

**Figure 3:**
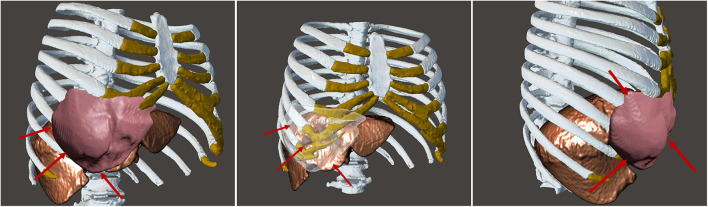
Final three-dimensional model and rendering from different perspectives showing the shape and consistency of the tumor and how the tumor is not invading the liver, a pivotal point for the surgery. The red arrows are pointing to the desmoid tumor to highlight the complete non-invasion of the liver and the circumscription of the tumor.

**Figure 4:**
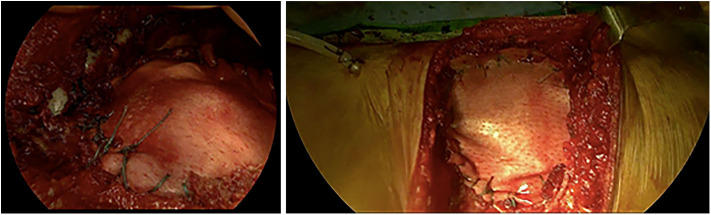
Intraoperative application of the Permacol™ patch fixed to the diaphragm (on the left) and the costal stumps (on the right).

A life-sized artificial model of the entire upper body was developed. In this case, CT scans were used to aid in the definition of the arterial and venous systems, tumor thrombus, and the boundary of the graft. As a best practice, a high-slice CT or magnetic resonance imaging scan should be used. For CT scans, we recommend slices of 1.25 mm, as used in this case, or lower. This process begins by importing the patient-specific Digital Imaging and Communications in Medicine (DICOM) data to Simpleware ScanIP software (Synopsis, Mountain View, CA, USA). The initial segmentation process involves defining the Hounsfield unit range for the anatomical structures (Figure 2) (Breakey et al., 2017). Using the threshold tool, the user can preview a mask that is overlaid on the scan slices to verify the units selected before applying the mask on all the scan slices. When the semiautomated masks have been applied to the aforementioned anatomical areas of the CT scan, the manual process of verifying the selection and modifying the masks to account for areas of variable signal or artifact begins. After verifying the masks for each anatomical region, the mask can be rendered into a 3D mesh. The mesh can be exported to specific file formats for various purposes. The industry standard for a robust multipart surface mesh is.stl (an abbreviation of stereolithography) file format. This.stl file can then be imported into a third software such as Autodesk Meshmixer (free product developed by Autodesk®, San Rafael, CA, USA) to add any labels or patient-specific identifiers. After the automatic segmentation, we refined the entire model by switching to manual segmentation (Marinozzi et al., 2013). The final 3D model (Figure 3) was evaluated by the surgical team in conjunction with the scans (Di Carlo et al., 2017). Based on the results of the model, the multidisciplinary team decided that the tumor was potentially resectable, and a surgical plan was developed to perform a complete resection based on the 3DR model, which was then discussed with the patient. We performed a right anterolateral thoracotomy on the seventh intercostal space at the clinically palpable lesion level. After entering the pleural cavity, it was confirmed that the neoplasm had invaded the 7th, 8th, 9th, and 10th anterior costal arches, some fibers of the overlying dentate muscle, and part of the diaphragm. En bloc resection of the diaphragm and thoracic wall invaded by the neoplasm was performed. The resulting diaphragmatic hole was repaired using a Permacol™ patch fixed with monofilament synthetic absorbable interrupted stitches (Ethibond Excel® 1/0) leaving a drainage catheter in the abdomen. To restore the chest wall support, a tailored 3DR-based patch cut out from a Permacol™ collagen-based implant (20 cm × 40 cm × 1.5 cm) was used. The patch was fixed to the adjacent ribs to cover the chest wall defect using monofilament synthetic (Ethibond Excel® 0/0) absorbable interrupted stitches (Figure 4) to provide both a barrier to prevent fluids and air from passing the pleura to the subcutaneous tissue and a scaffold supporting connective tissue regeneration. Additional soft tissue reconstruction was not required.

## Discussion

Chest wall resection for desmoid tumors can cause extensive chest wall defects. As evidenced by the variety of surgical techniques and treatment algorithms in the literature, the resulting defect remains a challenge. The fundamental goals of chest wall reconstruction are to fill all the dead space, restore the rigidity of the chest wall, preserve the lung function, protect the intrathoracic organs, provide a cover using soft tissue, minimize deformities, and allow patients to receive a radiotherapy when required. Therefore, a multidisciplinary approach is recommended, including the input of thoracic plastic and neurosurgeons, oncologists, radiotherapists, and bioengineers. Traditionally, a synthetic mesh and a musculocutaneous flap have been used to bridge the chest wall defect. However, the risk of secondary prosthesis infection exists. In this case, for the first time, we used a Permacol™ patch fitted to the chest wall defect, thanks to 3DR modeling that predicted the extent of bony resection. Permacol™ is a sterile sheet of acellular matrix that can provide the desired support and reduce the occurrence of complications from non-absorbable implants. Due to its chemical–physical features (providing strength and malleability) and its biocompatibility, Permacol™ is usually used for abdominal wall hernia repair and reconstruction or diaphragmatic defects (Limura and Giordano, 2017) but has also been proven to be successful when used in thoracic surgery (Mirzabeigi et al., 2011; D'Amico et al., 2018). Permacol™ guarantees both good malleability and stability in order to be incorporated into the host tissue with associated cell and microvascular ingrowth. At this level, the ribs have a “bucket handle” movement, i.e., expanding the chest increases the anteroposterior diameter. Although Permacol™ does not meet all the characteristics of an ideal material for rebuilding the chest wall because of the need to preserve the movement of the rib cage and the diaphragm, in this case, we preferred a certain elasticity at the expense of excessive rigidity, and Permacol™ achieved a good result as confirmed by a follow-up CT scan at 6 months showing no recurrences. A recent development is a CT scan with reconstructed 3D images, which could guide the production, via 3D printing technology (Bici et al., 2018; Tracy et al., 2019), of an accurate resin, polymer, metal, or degradable biomaterial prosthesis. The objectives of 3DR are the complete reproduction of sternal rib ends, maintenance of the intercostal space permeability, and reproduction of the shape of the resected specimen. Therefore, taking this case as a model, we developed a chest wall reconstruction plan using 3DR modeling.

## Conclusions

This case illustrated the potential power of 3D technology to enable a personalized approach to decision-making for better management of surgery. Specifically, this case illustrated that 3DR models may enable a personalized approach to the treatment of desmoid tumors, and this approach should be developed further and studied systematically. To construct an implant that resembles the human chest wall as possible, the next step will be selecting the best material type for reconstructing the chest wall. The optimal material should have the following characteristics: biocompatibility, strength as well as flexibility, and good resistance to infection.

## Data Availability Statement

The datasets generated for this study are available on request to the corresponding author.

## Ethics Statement

Ethical review and approval was not required for the study on human participants in accordance with the local legislation and institutional requirements. The patients/participants provided their written informed consent to participate in this study.

## Author Contributions

We adopt the CASRAI Contributor Roles Taxonomy (CRediT) for attribution (Brand et al., 2015). FM: conceptualization, funding acquisition, project administration, supervision, and writing—review and editing. FC: conceptualization, data curation, investigation, resources, validation, writing—original draft, and writing—review and editing. SN: conceptualization, data curation, investigation, methodology, visualization, writing—original draft, and writing—review and editing. MD and GC: conceptualization, resources, validation, and writing—review and editing. LP and FB: conceptualization, supervision, and writing—review and editing: All authors contributed to the article and approved the submitted version.

## Conflict of Interest

The authors declare that the research was conducted in the absence of any commercial or financial relationships that could be construed as a potential conflict of interest.
